# The Characterization of *Melanaphis sacchari* Microbiota and Antibiotic Treatment Effect on Insects

**DOI:** 10.3390/insects14100807

**Published:** 2023-10-11

**Authors:** Beatriz Xoconostle-Cázares, José Abrahán Ramírez-Pool, Leandro Alberto Núñez-Muñoz, Berenice Calderón-Pérez, Brenda Yazmín Vargas-Hernández, Rafael Bujanos-Muñiz, Roberto Ruiz-Medrano

**Affiliations:** 1 Departamento de Biotecnología y Bioingeniería, Centro de Investigación y de Estudios Avanzados Av., Instituto Politécnico Nacional 2508, Mexico City 07360, Mexico; bxoconos@cinvestav.mx (B.X.-C.); jramirezp@cinvestav.mx (J.A.R.-P.); leandro.nunez@cinvestav.mx (L.A.N.-M.); bcalderon@cinvestav.mx (B.C.-P.);; 2 Instituto Nacional de Investigaciones Forestales, Agrícolas y Pecuarias, Carretera Celaya-San Miguel de Allende km 6.5, Celaya Guanajuato 38110, Mexico; bujanos@live.com.mx

**Keywords:** *Melanaphis sacchari*, microbiota, antibiotics, insect control, *Wolbachia*

## Abstract

**Simple Summary:**

Insects are rapidly adapting to novel niches, such as crops. This is the case of the sugarcane aphid, a pest from Africa that is currently spreading worldwide. In the absence of males, aphids asexually generate many females, causing important economic losses for farmers. The microbiota in the insect was characterized and the presence of *Wolbachia* was also reported, correlating with its asexual reproduction. Antibiotic treatment of the aphid population resulted in a decrease in its survival rate. The possibility of controlling this pest by altering its microbiota is proposed in the present work.

**Abstract:**

Insects are under constant selective pressure, which has resulted in adaptations to novel niches such as crops. This is the case of the pest *Melanaphis sacchari*, the sugarcane aphid, native to Africa and currently spreading worldwide. The aphid undergoes successful parthenogenesis, causing important damage to a variety of crops and leading to important economic losses for farmers. A natural *M. sacchari* population grown in sorghum was studied to identify its microbiome through the sequencing of its 16S rDNA metagenome. A high proportion of Proteobacteria, followed by Firmicutes, Bacteroidetes, and Actinobacteria, was observed. We also detected *Wolbachia*, which correlates with the asexual reproduction of its host. *M. sacchari* was challenged in a bioassay with the antibiotics oxytetracycline and streptomycin, resulting in a dose-dependent decay of its survival rate. The possibility of controlling this pest by altering its microbiota is proposed.

## 1. Introduction

Aphids (Hemiptera: Aphididae) feed on the plant phloem sap, challenging photoassimilate allocation and causing stunted growth and yellowing, resulting in a significant impact on plant yield. Additionally, they are vectors for a diverse array of plant pathogenic viruses [1,2]. *Melanaphis sacchari* Zehntner (Hemiptera: Aphididae), the sugarcane aphid, is distributed worldwide and considered a major pest of sorghum (*Sorghum bicolor* (L.) Moench) and sugarcane (*Saccharum officinarum* L.). Furthermore, it has been reported on several important Poaceae crops such as wheat (*Triticum aestivum* L.), oat (*Avena sativa* L.), barley (*Hordeum vulgare* L.), rice (*Oryza sativa* L.), corn (*Zea mays* L.), and Johnson grass (*Sorghum halepense* (L.) Pers.), among others [3,4]. This aphid was first detected in the United States in the late 20th century. However, at that time, it was only considered a pest of sugarcane [5]. In 2013, a massive outbreak of *M. sacchari* occurred in sorghum, resulting in significant economic damage to crop growers in North America [5,6]. Nowadays, the sugarcane aphid has a global distribution, including the Americas, where the lack of natural predators makes its control more difficult [6,7]. Currently, chemical pesticides are employed for pest control, and other approaches include the development of aphid-resistant germoplasm [8]. Another strategy for controlling this pest has been the use of the predatory ladybugs (*Coccinella septempunctata*, Insecta: Coleoptera: Coccinellidae) and lacewings (*Chrysoperla* sp. (Neuroptera: Chrysopidae) as biological controls, with limited success due to the high reproductive rate of these insect species. Indeed, the most devastating impacts of the sugarcane aphid stem from its ability to rapidly reproduce and spread. *M. sacchari* asexual reproduction is favored under current crop culture. However, *M. sacchari* has shown sexual forms in Tamaulipas, Mexico, identified as winged adults with an average lifespan of 7.5 days, giving rise to 10.6 nymphs per female [9]. This reproductive success may be influenced by the insect microbiota, which has a symbiotic relationship with the host, providing essential nutrients by assimilating and degrading complex molecules present in the phloem sap from which it feeds [10,11]. The elimination of endosymbiotic and gut bacteria has been shown to provoke aphid death in *Pentalonia nigronervosa*, which is associated with the bacteria *Buchnera aphidicola* and *Wolbachia* sp. [12]. *Wolbachia* is a bacterium infecting the soft tissues of arthropods and is maternally inherited and involved in feminization and parthenogenesis. *Wolbachia*-infected insect females can asexually reproduce from unfertilized eggs, producing more females as offspring. This reproductive strategy is successful for *M. sacchari*, as the aphid population can be doubled in 1.7 days [13,14]. To profile the insect microbiota, massive nucleic acid analysis is an approach widely used, as no bacterial culture is needed. Among the available sequencing methods, nanopore sequencing (Oxford Nanopore Technologies, ONT) allows rapid library preparation and real-time data acquisition and has been employed to identify and profile bacterial communities [15]. ONT platforms such as MinION generate long sequences that allow complete coverage of the full-length 16S rRNA gene (V1-V9 regions) in a fast, cost-effective, and high-throughput manner [16]. The most relevant advantage of these full-length sequences is their high taxonomic (at least at species-level resolution) and phylogenetic resolution for bacterial identification, as they consider all informative sites of the 16S rRNA [17,18]. Therefore, *M. sacchari* microbiota diversity was assessed using this approach. We identified *Wolbachia* in these populations by PCR. The effect of the addition of antibiotics on *M. sacchari* was also evaluated. The use of antibiotics in bioassays significantly decreased nymph emergence, eventually resulting in its death, in a dose-dependent manner. The control of *M. sacchari* could thus be carried out employing antibiotics directed against bacteria, likely targeting its microbiota, including *Wolbachia*.

## 2. Materials and Methods

### 2.1. Establishment and Maintenance of M. sacchari Colonies

*M. sacchari* adults were collected from naturally infested sorghum at the National Institute of Agricultural and Livestock Forestry Research (INIFAP), Celaya Experimental Station, Celaya, Guanajuato, Mexico (20.581614, −100.822300) in December 2019. Fully expanded leaves containing all the insect developmental stages were carefully removed from 20 plants and transferred to an aphid growth chamber. The insect colonies were maintained at 28–30 °C with 16/8 h photoperiod (to mimic natural light conditions) using aphid-susceptible sorghum (*S. bicolor* cv. Sumiel II, Genex, Mexico). Leaf circles were placed in 80 mm Petri dishes containing 15 mL of 0.8% agar to create a humid environment. *M. sacchari adults* were then placed on the surface of the sorghum leaves in Petri dishes, and their activities and emergence of immature stages were monitored daily.

### 2.2. Massive Sequencing and 16S rRNA Analysis

Total DNA was purified from *M. sacchari* adults using Biopure Total DNA Extraction kit (Biopure, Mexico City, Mexico). Approximately one hundred adults collected from sorghum plants were rinsed with distilled water, homogenized with a sterile pestle, and the purified DNA was used for bacterial 16S rRNA gene amplification. The long-read 16S rRNA region was amplified with the primer set 27F (5′-AGAGTTTGATCMTGGCTCAG-3′) and 1492R (5′-TACGGYTACCTTGTTACGACTT-3′) [19], and PCR was performed with Takara Ex Polymerase (TakaraBio, Shiga, Japan) following the manufacturer’s recommendations. Fifty ng of genomic DNA and a final volume of 25 μL for each reaction were used. The PCR was performed under the following conditions: 94 °C for 5 min; 30 cycles of 30 s at 94 °C, 30 s at 55 °C, and 90 s at 72 °C; and a final extension at 72 °C for 10 min. The amplicons were then subjected to massive sequencing using Nanopore technology (Nanopore, Oxford, UK). Adapter ligation and purification were performed using the Ligation Sequencing Kit 1D (Oxford Nanopore Technologies, Oxford, UK) and Library Loading Bead Kit (Oxford Nanopore Technologies, Oxford, UK) following the manufacturer’s instructions. The amplicons were purified with the AMPure XP kit (Beckman Coulter, CA, USA) and quantified in a Qubit Fluorometer (Thermo Fisher Scientific, Waltham, MA, USA). Library preparation was carried out according to the manufacturer’s recommendations. The sequencing was performed on a MinION Mk1B nanopore sequencer (Oxford Nanopore Technologies, Oxford, UK) using a Flonge flow cell R9.4.1 (Oxford Nanopore Technologies, Oxford, UK).

Base-calling and adapter/barcode trimming were performed using Guppy software V.5.0.11 (Oxford Nanopore Technologies, Oxford, UK) to generate FASTQ-formatted sequence files. Additional filtering and taxonomic assignment were conducted using the NanoGalaxy platform [20], a Galaxy-based toolkit for long-read sequencing data, such as full-length 16S rRNA [21]. All tools were used with default parameters unless specified otherwise. The sequences were processed with porechop [22] (v.0.2.4, --discard_middle) and fastp [23] (v.0.23.2, --cut_by quality 9, −l200, --length_limit 2000), while taxonomic assignment was performed with kraken2 [24] (v.2.1.1, --db k2_standard_20210517, --confidence 0.01, --minimum-hit-groups 1). Additionally, abundances at genus and species levels were re-estimated with Bracken v.2.8 [25] (−t 1). Abundance re-estimated data were further visualized with Pavian [26] using the Pavian Metagenomics Data Explorer (https://fbreitwieser.shinyapps.io/pavian/#, access date: 30 July 2023). Alpha diversity indices were calculated with krakentools and the R packages vegan and fossil [27,28].

The quality-filtered FASTQ file was retrieved, and nucleotide sequences were extracted to obtain a FASTA file. Additionally, a database was constructed using available 16S sequences of *Wolbachia* spp., and a local nucleotide alignment was performed (BLAST 2.12.0+) with an E-value of 1 × 10^−6^ (https://ncbiinsights.ncbi.nlm.nih.gov/2021/07/09/blast-2-12-0/, access date: 5 August 2023).

### 2.3. Detection of Wolbachia in the M. sacchari Microbiome

Full-length metagenomic analysis of *M. sacchari* 16S microbiota showed low abundance reads taxonomically assigned to *Wolbachia*. To confirm this finding, *Wolbachia*-specific PCR primers (Table S1) were employed [29,30,31,32]. For amplification, 100 ng of purified DNA was used as template to target fragments of the tubulin polyglutamylase complex subunit 2 (*tpgs2*) gene from *M. sacchari*, along whit three fragments targeting *Wolbachia* genes: 16S ribosomal RNA gene (*wspec*), filamenting temperature-sensitive mutant Z (*ftsZ*), and outer surface protein (*wsp*81/691) [29,30,31,32]. PCR was carried out using a T100 Thermal cycler (Bio-Rad, Hercules, CA, USA) with the following conditions: an initial denaturation of 3 min at 94 °C, followed by 40 cycles of 30 s at 94 °C, 30 s at 60 °C, and 40 s at 72 °C, and a final extension of 5 min at 72 °C. The PCR products were subjected to Sanger sequencing at Macrogen Inc. (Seoul, Republic of Korea), and the resulting sequence reads were subjected to BLAST analysis against the NCBI database.

### 2.4. Antibiotic Treatment and Assessment of M. sacchari Viability

Oxytetracycline and streptomycin (Sigma Merck, Toluca, Mexico) were prepared at log concentrations of 0.1–10,000 μg/mL for each antibiotic. Antibiotic solutions were applied by spraying onto detached leaves that had been previously cut to fit the Petri dishes. Each side of the leaf was treated with 0.25 mL of the antibiotic solution, totaling 0.5 mL per leaf. After spray absorption, five active adults were placed on the treated leaves. As positive controls, the chemical pesticide abamectin (Agrimec 1.8%, Syngenta) was used at concentrations of 0.18%, 0.018%, and 0.0018%. Distilled water was used as a negative control. Each experiment was performed in triplicate. Aphids were monitored daily, and mortality was recorded when they detached from the leaves and changed color from brownish to black, remaining immobile. The offspring were identified by observing the nymphs and adults on the leaf surface using a stereoscope.

### 2.5. Statistical Analysis

Dead *M. sacchari* were counted for each treatment. Lethal concentration 50 (LC_50_) was calculated using linear regression and PROBIT software from Excel (Microsoft 365 MSO).

## 3. Results

### 3.1. The Establishment of M. sacchari Colonies in Detached Sorghum Leaves

The insect colony was reared within growth chambers designed to replicate the environmental conditions prevalent in sorghum fields. The *M. sacchari* colony dwelling on sorghum successfully completed its life cycle, which was evident through the emergence of adults alongside their immature nymphal stages. Within five days, the detached leaf retained its turgidity and verdant hue when housed in a humidified chamber. Despite their initial positioning on the adaxial leaf surface, the insects exhibited migratory behavior toward the abaxial side, analogous to their natural habitat across the entire plant. This migration, although consistent with their behavior in the field, remained worthless to the experimental setup as the antibiotic treatment was applied to both leaf surfaces. In the natural ecosystem, these insects reside on the abaxial side of the leaves, utilizing their stylets to suck the sap from the sorghum phloem. In our experiment, aphids capable of transitioning to the abaxial side were selected, whereas those residing on the adaxial surface were excluded in alignment with their typical distribution. Mortality occurred five days after the onset of antibiotic treatment, coinciding with the time frame of leaf viability in the in vitro aphid colony culture.

### 3.2. Biodiversity of M. sacchari Microbioma

**Figure 1:**
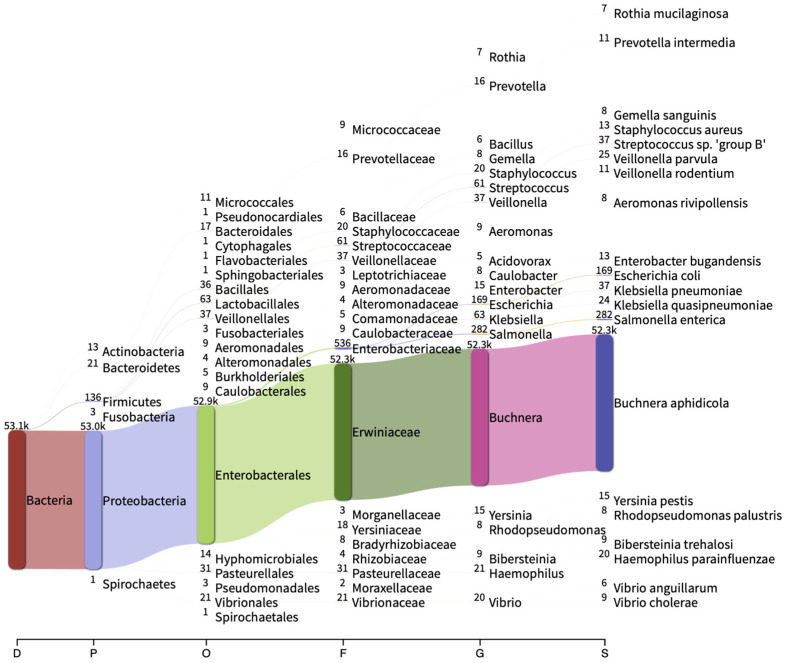
Sankey diagram showing the 20 most abundant taxa at different taxonomic levels. D: domain; P: phylum; O: order; F: family; G: genus; S: specie. The number of assigned reads for each taxon is showed to the left of each taxa.

The microbial composition of *M. sacchari* collected from commercially grown sorghum was characterized by 16S rRNA sequencing. Nanopore sequencing yielded 118,936 raw sequences (83.8 Mbp). After filtering, 53,273 sequences (73.5 Mbp) were retained, with 53,186 sequences (99.8%) successfully assigned to the Bacteria kingdom. Proteobacteria (99.670%), Firmicutes (0.256%), Bacteroidetes (0.040%), Actinobacteria (0.024%), Fusobacteria (0.006%), and Spirochaetes (0.002%) were identified at the phylum level. At the species level, *Buchnera aphidicola* (98.36%), *Salmonella enterica* (0.52%), and *Escherichia coli* (0.31%) were the most abundant (Figure 1). The OTUs exhibited relative abundances below 0.1% (Table S2), encompassing previously reported aphid endosymbionts such as *Acidovorax*, *Acinetobacter*, *Bacillus*, *Blochmannia*, *Candidatus* Ishikawaella, *Serratia*, *Sodalis*, *Sphingomonas*, and *Wolbachia*.

The species accumulation curve exhibits logarithmic behavior with a slope value close to zero, implying adequate coverage of the microbial community (Figure S1A). A total of 109 OTUs were identified, with approximately 60% consisting of low abundance reads, including singletons and doubletons. The calculated Good’s coverage index value indicated a comprehensive detection of most species within the community, suggesting adequate sampling and an accurate portrayal of the microbial diversity (Table 1). The ACE, Chao2, Jacknife1, and Jacknife2 estimators’ values indicate a high expected richness in the *M. sacchari* microbiota. On the other hand, when evaluating the evenness of abundance distribution using the Pielou J index, a value of 0.03 was observed, indicating uneven distribution of abundances in the community. Additionally, the Berger–Parker and Simpson indices indicated a high dominance of a few species in the bacterial community, whereas the abundances of the rest of the species were low. These findings were consistent with the species distribution graph (Figure S1B) and with the low Shannon index value, which considers both species richness and evenness in a sample or community.

### 3.3. Identification of Wolbachia in the M. sacchari Natural Population

**Figure 2:**
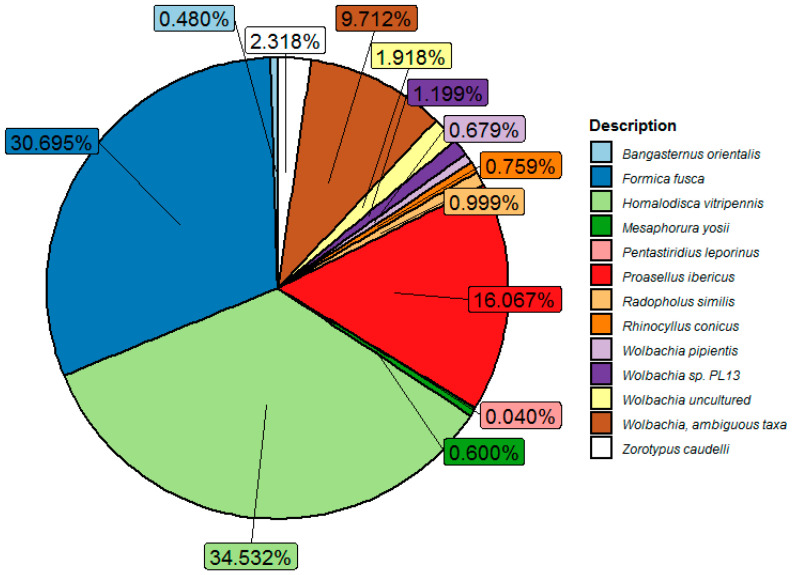
*Wolbachia* diversity in *M. sacchari* natural population identified in the Silva 16S rDNA V3 database. Sharpshooter *Homalodisca vitripennis* (ID JJNS01219994.16220.17700), followed in abundance by *Wolbachia* reported in *Formica fusca* (ID LI056258.85.1571) and *Wolbachia* sequences reported in *Proasellus ibericus* (ID HAFA01076328.88.1570), an isopod endemic to Slovenia. *W. pipientis* (MJMG01000007.69715.71219) infecting *Aedes albopictus*. *Wolbachia* infecting the nematode *Radopholus similis* (EU833482.1.1494), the hemipteran *Pentastiridius leporinu* (FN428797.1.1460), the hemimetabolous *Zorotypus caudelli* (GAYA02037411.2.1423), the springtail *Mesaphorura yosii* (KT799588.1.1420), the beetles used as biological control *Bangasternus orientalis* (M85266.1.1321), and *Rhinocyllus conicus* (M85267.1.1464) were also identified.

Parthenogenesis in *M. sacchari*, resulting in female colonies in sorghum, raised the possibility of the presence of *Wolbachia*, as it was described to be associated with sexual reproduction in different insect species. A metagenomic analysis of full-length 16S rDNA identified reads taxonomically assigned to *Wolbachia*. The *Wolbachia* sequences identified in the microbiome of *M. sacchari* showed a complex composition of 31 OTUs with different abundances. Figure 2 shows the counts of *Wolbachia* sequences previously reported in the sharpshooter *Homalodisca vitripennis*, followed in abundance by *Wolbachia* reported in *Formica fusca* and *Wolbachia* sequences reported in *Proasellus ibericus*, an isopod endemic to Slovenia.

Likewise, *W. pipientis* infecting *Aedes albopictus* and nematodes sequences were also identified. *Wolbachia* infecting the nematode *Radopholus similis*, the hemipteran *Pentastiridius leporinu*, the hemimetabolous *Zorotypus caudelli*, the springtail *Mesaphorura yosii*, the beetles used as biological controls, *Bangasternus orientalis*, and *Rhinocyllus conicus* were also identified.

**Figure 3:**
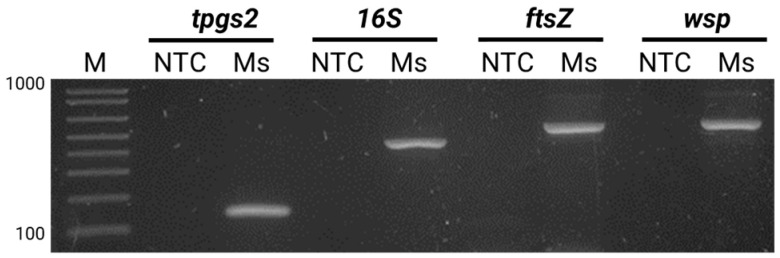
PCR detection of *Wolbachia* spp. in *M. sacchari* using specific primers against *M. sachhari* genome (*tpgs2*) and *Wolbachia* (*16S*, *ftsZ* and *wsp*). Amplified products were separated by electrophoresis on a 1% agarose gel. NTC: non-template control, Ms: *Melanaphis sacchari* pooled sample. M: 1 kb plus molecular weight marker (Invitrogen, Waltham, MA, USA).

To validate this finding, a PCR was performed using specific primers for three bacterial loci: *wspec*, *ftsZ*, and *wsp*81/91 (Figure 3). The resulting PCR products were purified, sequenced, and subjected to a BLAST analysis against GenBank, which confirmed the identity of the amplified products as the *Wolbachia* genome. The *wsp* (WSP81/691) amplicon showed 90.89% homology with 12 *Wolbachia* sp. accessions (MK053760.1, OQ102158.1, OQ102152.1, OQ102146.1, CP096926.1, CP096925.1, CP084693.1, CP067976.1, JX669538.1, EU499319.1, U83090.1, L02888.1). Similarly, the *16S* (WSPEC) amplicon, showed 97.44% homology with four *Wolbachia* sp. accessions found as insect endosymbionts of *Bactrocera dorsalis* (MK053760.1), *Paracorethrura iocnemis* (OQ102158.1), *Malenia bosnica* (OQ102152.1), and *Pentastira rorida* (OQ102146.1). Additionally, the *ftsZ* amplicon showed 99.81% homology with five *Wolbachia pipiens* accessions (HQ843850.1, JN316215.1, JN316214.1, JN316212.1, and JN316210.1). Furthermore, *tpgs2* amplicon sequence analysis confirmed host identity as *M. sacchari* (XM_025335778.1) with 100% homology.

### 3.4. Evaluation of Treated M. sacchari with Antibiotics

**Figure 4:**
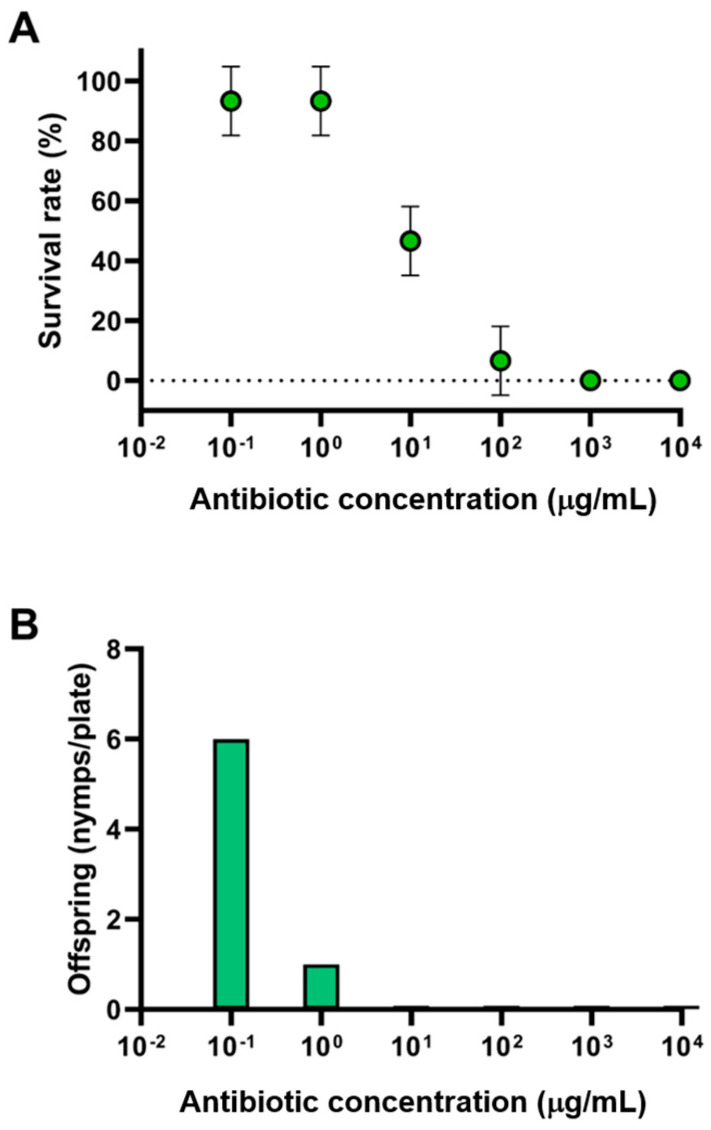
Effect of antibiotic concentration on the survival rate and offspring of *M. sacchari.* (**A**) Survival rate of *M. sacchari* exposed to different antibiotic concentrations. (**B**) Average offspring of *M. sacchari* exposed to different antibiotic concentrations.

The manipulation of microbial symbionts could alter insect fitness, e.g., by reducing co-metabolism or altering the host range or tolerance of abiotic conditions [33]. To evaluate the effect of antibiotics on aphid fitness, oxytetracycline and streptomycin were selected since they are currently employed in the integrated management of bacterial diseases of crop plants [34]. Tetracycline and its derivatives inhibit protein synthesis by preventing the attachment of aminoacyl-tRNA to the ribosomal acceptor site [35]. While streptomycin irreversibly binds to the 16S rRNA and S12 protein within the bacterial 30S ribosomal subunit, thus interfering with the initiation complex between mRNA and the bacterial ribosome. Six serial dilutions of oxytetracycline and streptomycin from 0.1, 1, 10, 100, 1000, and 10,000 μg/mL were evaluated. Sorghum leaves were placed into Petri dishes and kept turgid by the presence of an agar–water film. Adult mortality and count of *M. sacchari* offspring were evaluated by day 5, in which they would have reproduced at least once and the leaves were still turgent. The criterion for considering *M. sacchari* dead was that they remained motionless, frequently detached from the leaf surface, and in some cases, a black color developed on their body. In the control trials without antibiotics, the presence of offspring was recorded from the third day of incubation (Figure 4). The obtained values were used to calculate the linear regression and a PROBIT analysis, which determined the lethal concentration 50 (LC_50_) at 6.6 μg/mL (Table S3).

Higher concentrations of antibiotics produced death in the entire aphid population, demonstrating that aphid mortality is dose-dependent. In a parallel test, the chemical insecticide abamectin was used as a positive control to demonstrate the susceptibility of the insects under the assayed conditions. Concentrations of 1.8, 0.18, and 0.018 μg/mL of the active ingredient abamectin were used, registering 100% mortality in all tested concentrations.

## 4. Discussion

The sugarcane *M. sacchari* has a worldwide distribution, and it has recently adapted as a pest of sorghum, severely damaging its production [5,36]. In general, aphids are one of the most persistent threats to agriculture given their ability to feed on phloem sap from plants, which greatly compromises the ability of plants to translocate photosynthates from source to sink tissues. Another threat posed by aphids is that they are vectors for a great variety of viruses [2]. The notable adaptability of aphids to colonize novel hosts challenges the defense mechanisms against insect feeding developed by plants throughout evolution and can be attributed, at least in part, to their reproductive success. In turn, this involves the continuous shift from sexual to asexual reproduction and interaction with other agents such as viruses [37]. There is a high incidence of asexual reproduction in agricultural pests, aphids among them [38]. While parthenogenesis may lead to a decrease in natural variation within a given population, this strategy results in rapid reproduction and propagation, which may contribute to their persistence in certain conditions, such as those favored in constant environments afforded by monocultures [38]. In fact, it is known that parthenogenesis in several insects feeding on plants is caused by the presence of the endosymbiont bacterium *Wolbachia* [39].

Plants have evolved a range of defense mechanisms to protect themselves from herbivory, including the production of antifeeding compounds that reduce the nutritional value of plant tissues for herbivores, like sap-feeding insects. Ecologically, these compounds play an important role in shaping plant–herbivore interactions, thus reducing crop damage. However, the insect microbiota’s co-metabolism overcomes those limitations by successfully degrading complex compounds [11]. The microflora of insects consists of a diverse community of microorganisms that can break down a wide range of complex molecules, including antinutrient compounds such as phytic acids, tannins, polyphenols, enzyme inhibitors, saponins, and lectins. Therefore, the absence of a healthy microbiota could hamper insect development. The application of antibiotics to *M. sacchari* reproduction and survival was designed to target gut bacterial symbionts, including *Wolbachia*. A dose-dependent impact on reproduction and lifespan was observed in the five-day assays. As expected, in the control experiments without antibiotics, offspring were observed on the third day of incubation. This is consistent with extant observations [40], where antibiotic treatment against the brown planthopper (*Nilaparvata lugens*) increased host insecticide susceptibility via suppression of bacterial symbionts. In our study, the lowest antibiotic concentration of 0.1 μg/mL impacted reproduction, as no progeny was observed, while higher concentrations of antibiotics were lethal for the entire *M. sacchari* population. The impact on the reproductive rate could be due to the insect’s inability to feed and maintain its metabolism to produce progeny, although selective suppression of *Wolbachia* by antibiotic treatment as a control strategy needs to be further evaluated.

In the insect colony maintained on sorghum leaves in Petri dishes, the emergence of nymphs could be observed. This suggests that female reproduction was similar to what is observed in their natural environment; therefore, the in vitro assay was used to assess insect reproductive success and mortality. A limitation of the use of Petri dishes containing aphid colonies is that the leaf loses turgidity despite being in a humid atmosphere. Five-day trials were successfully evaluated. The insect behavior in this microenvironment was similar to that observed in sorghum plants in the field. The composition of the *M. sacchari* microbiome was analyzed through the amplification of the V3 fragment of the 16S rDNA using Nanopore equipment, long used to describe microbiomes in different systems [15,16]. Each different OTU was considered equivalent to an organism; thus, there is the possibility of overestimating the population present if there is more than one 16S rDNA gene in the genome of a microorganism. The sequencing of 118,835 purified OTUs and their comparison with existing databases allowed us to identify microorganisms belonging to Alpha proteobacteria, Firmicutes, Bacteroidetes, and Actinobacteria. These taxa are well represented in the microbiomes of other insects and are part of their gut flora [41]. Different families like Bacteroidales, Enterobacteriales, Bacillales, Lactobacillales, Actinomycetales, Pasteurellales, Rickettsiales, and Rhodospirillales were identified as previously described in other microbiomes. At the genus level, it was possible to identify *Buchnera*, *Staphylococcus*, *Prevotella*, *Bacillus*, *Streptococcus*, *Granulicatella*, *Pasteurella*, *Acetobacter*, *Rothia*, *Veillonella*, *Leptotrichia*, *Blastobacter*, *Clostridium*, *Desulfomicrobium*, *Bradyrhizobium*, and *Wolbachia*. *Wolbachia* has been detected before in sorghum in the US [36]. However, given that microbiota varies on a geographical basis in aphids [42], the bacterial populations within *M. sacchari* adapted to a sorghum variety widely used in central Mexico (Sumiel II) would not necessarily be the same. Indeed, previous reports on the microbiome of *M. sacchari* infesting sorghum grown in northeastern Mexico did not report the presence of *Wolbachia* [43], underscoring the need for sequencing diverse aphid microbiomes. Rarefaction curve showed a behavior that was asymptotic in the diversity graph [44], indicating a high diversity in the population of microorganisms present on the aphid.

The finding of sequences homologous to *Wolbachia* in the *M. sacchari* microbiome was later confirmed by amplifying three loci previously described for this genus: *wspec*, *ftsZ*, and *wsp*81/91. The homology of these sequences with the *Wolbachia* genome demonstrated its presence in aphids. As in other insects, *Wolbachia* could bias the reproductive strategy to parthenogenetic for its transmission. In this case, it would also be beneficial for the aphid since an adult produces 4–12 nymphs during its lifespan [5,9]. Parthenogenesis induced by *Wolbachia* has been observed in a range of insect species, including mites, fruit flies, and wasps [45]. However, its molecular basis is not well understood. Differential gene expression involved in the fertilization process as well as endoreduplication and chromosome duplication are some of the likely underlying causes [46].

It is not clear whether suppression of *Wolbachia* and other endosymbionts from the aphid microbiota could have an impact on the insect’s reproductive ability, although sublethal concentrations of tetracycline negatively affect the fecundity of the parasitoid *Trichogramma pretiosum* [47]. Similar results have been observed on the Coffee Berry Borer, lending credence to the notion that *Wolbachia* influences its host fecundity [48]. Given that the analysis of the microbiota of *M. sacchari* infesting Sumiel II sorghum revealed the presence of *Wolbachia*, the effect of this antibiotic on insect viability was evaluated.

From an applied viewpoint, the use of antimicrobial agents like antibiotics could help solve the problem of *M. sacchari* infestation in sorghum since it has no natural enemies and biological control has had limited success [49]. Despite these observed effects, potentially detrimental effects to beneficial organisms’ microbiota in the agroecosystem should be monitored. In addition, the possible impact on asexual reproduction needs to be assessed on the field to calculate the control or mitigation of the *M. sacchari* population in plants.

*M. sacchari* populations in this crop are permanent in agricultural areas, even when sorghum is not planted, because they have adapted to other crops, including weeds. Its persistence at low densities allows it to re-colonize sorghum when the crop cycle begins. In all, the potential alteration of pest insect or vector microbiota offers the possibility of reducing their impact on crops in an agroecological context.

## Figures and Tables

**Table 1:** Alpha diversity indices and measures of *M. sacchari* microbiota community.

Type	Index or Measure	Value
Observed Richness	OTUs richness	110.000
	Singletons	0.5090
	Dobletons	0.1273
	Good’s coverage	0.9989
Richness estimators	ACE	246.000
	Chao2 (S_chao_)	365.080
	Jacknife-1	270.998
	Jacknife-2	343.990
Evenness	Pielou J	0.0300
Dominance	Berger–Parker (d)	0.9860
	Simpson (D)	0.0279
Combined diversity	Shannon (H’)	0.1198

## Data Availability

Data sequences are available at SRA data: PRJNA966980.

## Supplementary Materials

The following supporting information can be downloaded at https://www.mdpi.com/article/10.3390/insects14100807/s1. Table S1. Primers used for amplification of target genes from *M. sacchari* and *Wolbachia* spp [29,30,31,32]. Table S2. Relative abundances of the *M. sacchari* microbiota. Table S3. Effect of oxytetracycline and streptomycin on *M. sacchari* survival rate. Figure S1. Rarefaction curve and abundance distribution in the microbial community of *M. sacchari*.
