# Supplementary material for: The Characterization of Melanaphis sacchari Microbiota and Antibiotic Treatment Effect on Insects
Source: Insects. 2023 Oct 11;14(10):807. doi: 10.3390/insects14100807 (PMC10607097; doi:10.3390/insects14100807)
Supplement: Supplementary file 1 [file insects-14-00807-s001.zip › insects-2625259-supplementary.pdf]

Supplementary material

Supplementary tables

Table S1. Primers used for amplification of target genes from *M. sacchari* and *Wolbachia* spp.

Gen	Organism	Primer Name	Sequence (5′→3′)	Amplicon (bp)	Reference
tpgs2	*M. sacchari*	MsTPGS2-F	TTGCCCATCGGAAACATCACTGTG	135	This work
		MsTPGS2-R	TGGCGTGTTCTTGTCGGATAAACG		
16S	*Wolbachia* spp.	WSPEC-F	CATACCTATTCGAAGGGATAG	438	[1]
		WSPEC-R	AGCTTCGAGTGAAACCAATTC		
ftsZ		Wolbachia FtsZ-F	TACTGACTGTTGGAGTTGTAACTAAGCCGT	570	[2]
		Wolbachia FtsZ-R	TGCCAGTTGCAAGAACAGAAACTCTAACTC		
wsp		Wolbachia sp-81F	TGGTCCAATAAGTGATGAAGAAAC	≈600	[3,4]
		Wolbachia sp-691R	AAAAATTAAACGCTACTCCA		

1. [1] Werren, J.H.; Windsor, D.M. *Wolbachia* Infection Frequencies in Insects: Evidence of a Global Equilibrium? *Proc. R. Soc. Lond. B Biol. Sci.* **2000**, *267*, 1277–1285. https://doi.org/10.1098/rspb.2000.1139.
2. [2] Zha, X.; Zhang, W.; Zhou, C.; Zhang, L.; Xiang, Z.; Xia, Q. Detection and Characterization of Wolbachia Infection in Silkworm. *Genet. Mol. Biol.* **2014**, *37*, 573–580. https://doi.org/10.1590/S1415-47572014000400014.
3. Zhou, W.; Rousset, F.; O'Neill, S. Phylogeny and PCR–based Classification of Wolbachia Strains Using Wsp Gene Sequences. *Proc. R. Soc. Lond. B Biol. Sci.* **1998**, *265*, 509–515. https://doi.org/10.1098/rspb.1998.0324.
4. Braig, H.R.; Zhou, W.; Dobson, S.L.; O'Neill, S.L. Cloning and Characterization of a Gene Encoding the Major Surface Protein of the Bacterial Endosymbiont *Wolbachia pipientis*. *J. Bacteriol.* **1998**, *180*, 2373–2378. https://doi.org/10.1128/JB.180.9.2373-2378.1998.

Table S2. Relative abundances of the *M. sacchari* microbiota.

Phylum	Class	Order	Family	Genus	Specie	Relative abundance
Proteobacteria	Gammaproteobacteria	Enterobacterales	Erwiniaceae	Buchnera	*Buchnera aphidicola*	0.98365
Proteobacteria	Gammaproteobacteria	Enterobacterales	Enterobacteriaceae	Salmonella	*Salmonella enterica*	0.00521
Proteobacteria	Gammaproteobacteria	Enterobacterales	Enterobacteriaceae	Escherichia	*Escherichia coli*	0.00314
Firmicutes	Bacilli	Lactobacillales	Streptococcaceae	Streptococcus		0.00070
Proteobacteria	Gammaproteobacteria	Enterobacterales	Enterobacteriaceae	Klebsiella	*Klebsiella pneumoniae*	0.00068
Firmicutes	Negativicutes	Veillonellales	Veillonellaceae	Veillonella	*Veillonella parvula*	0.00047
Proteobacteria	Gammaproteobacteria	Enterobacterales	Enterobacteriaceae	Klebsiella	*Klebsiella quasipneumoniae*	0.00045
Proteobacteria	Gammaproteobacteria	Pasteurellales	Pasteurellaceae	Haemophilus	*Haemophilus parainfluenzae*	0.00038
Proteobacteria	Gammaproteobacteria	Enterobacterales	Yersiniaceae	Yersinia	*Yersinia pestis*	0.00028
Proteobacteria	Gammaproteobacteria	Enterobacterales	Yersiniaceae	Yersinia		0.00028
Firmicutes	Bacilli	Bacillales	Staphylococcaceae	Staphylococcus	*Staphylococcus aureus*	0.00024
Proteobacteria	Gammaproteobacteria	Enterobacterales	Enterobacteriaceae	Enterobacter	*Enterobacter bugandensis*	0.00024
Bacteroidetes	Bacteroidia	Bacteroidales	Prevotellaceae	Prevotella	*Prevotella intermedia*	0.00021
Firmicutes	Negativicutes	Veillonellales	Veillonellaceae	Veillonella	*Veillonella rodentium*	0.00021
Proteobacteria	Gammaproteobacteria	Vibrionales	Vibrionaceae	Vibrio	*Vibrio cholerae*	0.00017
Firmicutes	Bacilli	Bacillales		Gemella	*Gemella sanguinis*	0.00015
Proteobacteria	Alphaproteobacteria	Hyphomicrobiales	Bradyrhizobiaceae	Rhodopseudomonas	*Rhodopseudomonas palustris*	0.00015
Proteobacteria	Gammaproteobacteria	Aeromonadales	Aeromonadaceae	Aeromonas	*Aeromonas rivipollensis*	0.00015
Proteobacteria	Gammaproteobacteria	Pasteurellales	Pasteurellaceae	Bibersteinia	*Bibersteinia trehalosi*	0.00015
Actinobacteria	Actinomycetia	Micrococcales	Micrococcaceae	Rothia	*Rothia mucilaginosa*	0.00013
Firmicutes	Bacilli	Bacillales	Bacillaceae	Bacillus		0.00011
Bacteroidetes	Bacteroidia	Bacteroidales	Prevotellaceae	Prevotella	*Prevotella melaninogenica*	0.00009
Proteobacteria	Betaproteobacteria	Burkholderiales	Comamonadaceae	Acidovorax		0.00009
Proteobacteria	Betaproteobacteria	Burkholderiales	Comamonadaceae	Acidovorax		0.00009

Phylum	Class	Order	Family	Genus	Specie	Relative Abundance
Proteobacteria	Gammaproteobacteria	Vibrionales	Vibrionaceae	Vibrio	*Vibrio anguillarum*	0.00009
Firmicutes	Bacilli	Lactobacillales	Streptococcaceae	Streptococcus	*Streptococcus dysgalactiae*	0.00008
Firmicutes	Bacilli	Lactobacillales	Streptococcaceae	Streptococcus		0.00008
Firmicutes	Bacilli	Lactobacillales	Streptococcaceae	Streptococcus	*Streptococcus thermophilus*	0.00008
Proteobacteria	Alphaproteobacteria	Caulobacterales	Caulobacteraceae	Caulobacter	*Caulobacter mirabilis*	0.00008
Proteobacteria	Alphaproteobacteria	Hyphomicrobiales	Rhizobiaceae	Liberibacter	*Candidatus Liberibacter asiaticus*	0.00008
Actinobacteria	Actinomycetia	Actinomycetales	Actinomycetaceae	Actinomyces		0.00006
Firmicutes	Bacilli	Bacillales	Bacillaceae	Bacillus	*Bacillus velezensis*	0.00006
Fusobacteria	Fusobacteriia	Fusobacteriales	Leptotrichiaceae	Leptotrichia	*Leptotrichia sp. oral taxon 212*	0.00006
Fusobacteria	Fusobacteriia	Fusobacteriales	Leptotrichiaceae	Leptotrichia		0.00006
Proteobacteria	Alphaproteobacteria	Caulobacterales	Caulobacteraceae	Caulobacter	*Caulobacter flavus*	0.00006
Proteobacteria	Alphaproteobacteria	Hyphomicrobiales	Bradyrhizobiaceae	Bradyrhizobium		0.00006
Proteobacteria	Gammaproteobacteria	Alteromonadales	Alteromonadaceae	Alteromonas	*Alteromonas sp. RW2A1*	0.00006
Proteobacteria	Gammaproteobacteria	Alteromonadales	Alteromonadaceae	Alteromonas		0.00006
Proteobacteria	Gammaproteobacteria	Vibrionales	Vibrionaceae	Vibrio	*Vibrio campbellii*	0.00006
Actinobacteria	Actinomycetia	Micrococcales	Microbacteriaceae	Microbacterium	*Microbacterium hominis*	0.00004
Firmicutes	Bacilli	Bacillales	Staphylococcaceae	Staphylococcus	*Staphylococcus sp. AntiMn-1*	0.00004
Firmicutes	Bacilli	Bacillales	Staphylococcaceae	Staphylococcus	*Staphylococcus warneri*	0.00004
Firmicutes	Bacilli	Lactobacillales	Streptococcaceae	Streptococcus	*Streptococcus parasanguinis*	0.00004
Firmicutes	Bacilli	Lactobacillales	Streptococcaceae	Streptococcus	*Streptococcus pyogenes*	0.00004
Firmicutes	Bacilli	Lactobacillales	Streptococcaceae	Streptococcus	*Streptococcus sp. 116-D4*	0.00004
Firmicutes	Bacilli	Lactobacillales	Streptococcaceae	Streptococcus	*Streptococcus sp. oral taxon 061*	0.00004
Proteobacteria	Betaproteobacteria	Neisseriales	Neisseriaceae	Neisseria		0.00004
Proteobacteria	Gammaproteobacteria	Enterobacterales	Bruguierivoracaceae	Sodalis	*Candidatus Sodalis pierantonius*	0.00004
Proteobacteria	Gammaproteobacteria	Enterobacterales	Enterobacteriaceae	Enterobacter	*Enterobacter ludwigii*	0.00004
Proteobacteria	Gammaproteobacteria	Enterobacterales	Hafniaceae	Edwardsiella	*Edwardsiella ictaluri*	0.00004

Phylum	Class	Order	Family	Genus	Specie	Relative abundance
Proteobacteria	Gammaproteobacteria	Enterobacterales	Morganellaceae	Proteus	*Proteus vulgaris*	0.00004
Proteobacteria	Gammaproteobacteria	Enterobacterales	Yersiniaceae	Serratia	*Serratia marcescens*	0.00004
Proteobacteria	Gammaproteobacteria	Vibrionales	Vibrionaceae	Vibrio	*Vibrio taketomensis*	0.00004
Actinobacteria	Actinomycetia	Micrococcales	Micrococcaceae	Arthrobacter		0.00002
Actinobacteria	Actinomycetia	Micrococcales	Micrococcaceae	Arthrobacter		0.00002
Actinobacteria	Actinomycetia	Pseudonocardiales	Pseudonocardiaceae	Amycolatopsis		0.00002
Actinobacteria	Actinomycetia	Pseudonocardiales	Pseudonocardiaceae	Amycolatopsis		0.00002
Bacteroidetes	Bacteroidia	Bacteroidales	Rikenellaceae	Alistipes		0.00002
Bacteroidetes	Bacteroidia	Bacteroidales	Rikenellaceae	Alistipes		0.00002
Bacteroidetes	Cytophagia	Cytophagales	Cytophagaceae	Rhodocytophaga	*Rhodocytophaga rosea*	0.00002
Bacteroidetes	Flavobacteriia	Flavobacteriales	Weeksellaceae	Chryseobacterium	*Chryseobacterium camelliae*	0.00002
Bacteroidetes	Flavobacteriia	Flavobacteriales	Weeksellaceae			0.00002
Bacteroidetes	Sphingobacteriia	Sphingobacteriales	Sphingobacteriaceae	Mucilaginibacter	*Mucilaginibacter gotjawali*	0.00002
Firmicutes	Bacilli	Bacillales	Bacillaceae	Bacillus	*Bacillus amyloliquefaciens*	0.00002
Firmicutes	Bacilli	Bacillales	Bacillaceae	Bacillus	*Bacillus cereus*	0.00002
Firmicutes	Bacilli	Bacillales	Bacillaceae	Bacillus	*Bacillus thuringiensis*	0.00002
Firmicutes	Bacilli	Bacillales	Listeriaceae	Listeria	*Listeria monocytogenes*	0.00002
Firmicutes	Bacilli	Bacillales	Staphylococcaceae	Staphylococcus	*Staphylococcus auricularis*	0.00002
Firmicutes	Bacilli	Bacillales	Staphylococcaceae	Staphylococcus	*Staphylococcus pettenkoferi*	0.00002
Firmicutes	Bacilli	Bacillales	Staphylococcaceae	Staphylococcus	*Staphylococcus pseudintermedius*	0.00002
Firmicutes	Bacilli	Lactobacillales	Carnobacteriaceae			0.00002
Firmicutes	Bacilli	Lactobacillales	Enterococcaceae	Enterococcus	*Enterococcus cecorum*	0.00002
Firmicutes	Bacilli	Lactobacillales	Streptococcaceae	Streptococcus	*Streptococcus iniae*	0.00002
Firmicutes	Bacilli	Lactobacillales	Streptococcaceae	Streptococcus	*Streptococcus mitis*	0.00002
Firmicutes	Bacilli	Lactobacillales	Streptococcaceae	Streptococcus	*Streptococcus periodonticum*	0.00002
Proteobacteria	Alphaproteobacteria	Caulobacterales	Caulobacteraceae	Brevundimonas		0.00002

Phylum	Class	Order	Family	Genus	Specie	Relative abundance
Proteobacteria	Alphaproteobacteria	Caulobacterales	Caulobacteraceae	Phenylobacterium	*Phenylobacterium parvum*	0.00002
Proteobacteria	Alphaproteobacteria	Hyphomicrobiales	Aurantimonadaceae	Aureimonas		0.00002
Proteobacteria	Alphaproteobacteria	Hyphomicrobiales	Bradyrhizobiaceae	Tardiphaga		0.00002
Proteobacteria	Alphaproteobacteria	Hyphomicrobiales	Brucellaceae	Brucella		0.00002
Proteobacteria	Alphaproteobacteria	Hyphomicrobiales	Brucellaceae	Brucella		0.00002
Proteobacteria	Alphaproteobacteria	Hyphomicrobiales	Brucellaceae			0.00002
Proteobacteria	Alphaproteobacteria	Hyphomicrobiales	Methylobacteriaceae			0.00002
Proteobacteria	Alphaproteobacteria	Rhodospirillales	Acetobacteraceae	Roseomonas		0.00002
Proteobacteria	Alphaproteobacteria	Rickettsiales	Anaplasmataceae	Wolbachia		0.00002
Proteobacteria	Alphaproteobacteria	Rickettsiales	Anaplasmataceae			0.00002
Proteobacteria	Alphaproteobacteria	Sphingomonadales	Sphingomonadaceae	Sphingomonas		0.00002
Proteobacteria	Deltaproteobacteria					0.00002
Proteobacteria	Gammaproteobacteria	Aeromonadales	Aeromonadaceae	Aeromonas	*Aeromonas veronii*	0.00002
Proteobacteria	Gammaproteobacteria	Alteromonadales	Alteromonadaceae	Marinobacter	*Marinobacter salsuginis*	0.00002
Proteobacteria	Gammaproteobacteria	Enterobacterales	Enterobacteriaceae	Candidatus Blochmannia		0.00002
Proteobacteria	Gammaproteobacteria	Enterobacterales	Enterobacteriaceae	Candidatus Blochmannia		0.00002
Proteobacteria	Gammaproteobacteria	Enterobacterales	Enterobacteriaceae	Candidatus Doolittlea	*Candidatus Doolittlea endobia*	0.00002
Proteobacteria	Gammaproteobacteria	Enterobacterales	Enterobacteriaceae	Candidatus Ishikawaella	*Candidatus Ishikawaella capsulata*	0.00002
Proteobacteria	Gammaproteobacteria	Enterobacterales	Enterobacteriaceae	Candidatus Riesia		0.00002
Proteobacteria	Gammaproteobacteria	Enterobacterales	Enterobacteriaceae	Cronobacter	*Cronobacter sakazakii*	0.00002
Proteobacteria	Gammaproteobacteria	Enterobacterales	Enterobacteriaceae	Klebsiella	*Klebsiella oxytoca*	0.00002
Proteobacteria	Gammaproteobacteria	Enterobacterales	Enterobacteriaceae	Klebsiella	*Klebsiella variicola*	0.00002
Proteobacteria	Gammaproteobacteria	Enterobacterales	Enterobacteriaceae	Raoultella	*Raoultella planticola*	0.00002
Proteobacteria	Gammaproteobacteria	Enterobacterales	Erwiniaceae	Erwinia	*Candidatus Erwinia haradaeae*	0.00002
Proteobacteria	Gammaproteobacteria	Enterobacterales	Morganellaceae	Providencia	*Providencia rettgeri*	0.00002
Proteobacteria	Gammaproteobacteria	Enterobacterales	Pectobacteriaceae	Dickeya	*Dickeya dadantii*	0.00002

Phylum	Class	Order	Family	Genus	Specie	Relative abundance
Proteobacteria	Gammaproteobacteria	Enterobacterales	Yersiniaceae	Serratia	*Serratia symbiotica*	0.00002
Proteobacteria	Gammaproteobacteria	Pasteurellales	Pasteurellaceae	Haemophilus	*Haemophilus pittmaniae*	0.00002
Proteobacteria	Gammaproteobacteria	Pasteurellales	Pasteurellaceae	Pasteurella	*Pasteurella multocida*	0.00002
Proteobacteria	Gammaproteobacteria	Pseudomonadales	Moraxellaceae	Acinetobacter	*Acinetobacter baumannii*	0.00002
Proteobacteria	Gammaproteobacteria	Pseudomonadales	Moraxellaceae	Acinetobacter	*Acinetobacter lanii*	0.00002
Proteobacteria	Gammaproteobacteria	Vibrionales	Vibrionaceae	Photobacterium	*Photobacterium damselae*	0.00002
Spirochaetes	Spirochaetia	Spirochaetales	Spirochaetaceae	Treponema	*Treponema parvum*	0.00002

Table S3. Effect of oxytetracycline and streptomycin on *M. sacchari* survival rate.

**Concentration (µg/mL)**	**Dead aphids in each replicate**	**Total**	**Percentage %**	**Offspring in 5 days**
0.0	0,0,0	0	0	5
0.1	1,0,0	1	6.6	6
1.0	1,0,0	1	6.6	1
10	3,3,2	8	53.3	0
100	5,4,5	14	93.3	0
1000	5,5,5	15	100	0
10000	5,5,5	15	100	0

Data transformation for linear regression analysis

**Concentration (µg/mL)**	**log10**	**Dead aphids (%)**	**Probit**
0	-	0	-
0.1	-1	6.6	3.45
1	0	6.6	3.45
10	1	53.3	5.08
100	2	93.3	6.48
1000	3	100	8.09
10000	4	100	8.09

Multiple regression coefficient = 0.97040289; R^2 = 0.94168178
Adjusted R^2 = 10222; Typical error = 0.57287163

Summary of statistics analysis by linear regression

	Degrees of freedom	*Sum of squares*	*Average of squares*	*F*	*Critic value of F*
Regression	1	21.1970057	21.1970057	64.589197	0.00130102
Residues	4	1.31272762	0.3281819		
Total	5	22.5097333			

	Coefficients	*Typical error*	*Statistical t*
Interception	4.12247619	0.311274478	13.2438619
Variable X 1	1.10057143	0.136942512	8.03674045

Probability	*Inferior limit for a confidence threshold=95%*	*Superior limit for a confidence threshold= 95%*
0.00018783	3.25823969	4.98671269
0.00130102	0.72035806	1.4807848

LC_{50}, employing y=mx+b: 0.81818182
Antilog= 6.57933225
Calculated LC_{50} = **6.6 µg/mL**

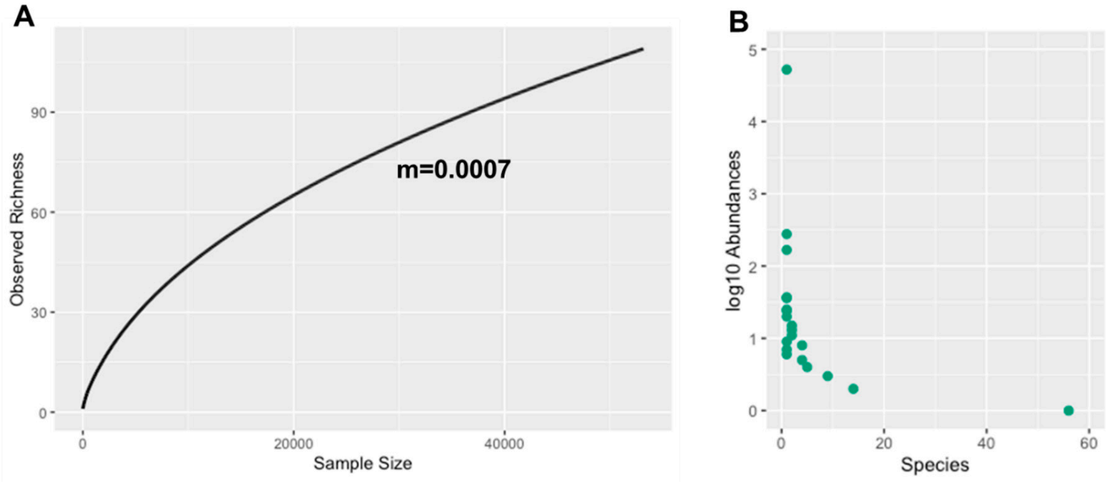

Figure S1. Rarefaction curve and abundance distribution in the microbial community of *M. sacchari***.** A) Accumulation of species curve showing the slope value (m) of the accumulation curve. B) Abundance distribution of the *M. sacchari* microbial community.
